# Nonirritant concentrations and amount of active ingredient in drug patch tests optimal concentrations for DPTs

**DOI:** 10.1186/2045-7022-4-S3-P1

**Published:** 2014-07-18

**Authors:** Delphine Brajon, Sophie Menetre, Julie Waton, Claire Poreaux, Annick Barbaud

**Affiliations:** 1 Department of Dermatology, Brabois Hospital, France; 2 Department of Pharmacy, Brabois hospital, France

## Background

Drug patch tests (DPTs) with medications suspected of causing an allergic reaction represent a method of diagnostic testing that is low risk, as DPTs can reproduce delayed hypersensitivity to drugs and entail only a moderate re-exposure of patients to potential offending drugs. We assessed the non-irritating concentration of DPTs to determine the amount of active ingredient (AI) contained in the drugs used in the tests.

## Method

From a retrospective, single-center study of all patients treated during a 6-year period for etiological investigations following a drug eruption, each potentially responsible drug was tested from the commercially available preparation diluted to 30% of its concentration in water, petrolatum, or alcohol. Data collection was performed with a customized computer database. For each type of DPT studied, the numbers of positive and negative tests were recorded. The amount of AI contained in the DPT (as a percentage) was then calculated after weighing each tablet.

## Results

Of the 5,558 DPTs studied, all were nonirritant. The average concentration of AI was 9.8%; 25% of DPTs had an AI concentration less than 2%, and 25% displayed an AI concentration above 16%. The AI concentration ranged from 0.05% (digoxin) to 30% (paracetamol lyophilisate).

## Discussion

The actual concentrations of the AI in DPTs done at 30% of the commercially available form varied from 0.05% to 27.08% when tablets were used. Any report of the results of a DPT must give the exact concentration of the AI applied.

## Conclusion

These data provide thresholds for the non-irritating concentration of 89 different drugs, and will help to standardize DPT methods.

